# Candidate imprinting control regions in dog genome

**DOI:** 10.1186/s12864-025-11801-9

**Published:** 2025-07-30

**Authors:** Phillip Wyss, Minou Bina

**Affiliations:** Department of Chemistry, Purdue University, West Lafayette, IN 47907 USA

**Keywords:** Allele-specific, *Canis familiaris*, Canine, CpG, Dog genome, DMR, ICR, Genome-wide, Gene regulation, Imprinted genes

## Abstract

In mammals, genomic imprinting restricts the expression of a subset of genes from one of the two parental alleles. The process is regulated by imprinting control regions (ICRs) dispersed across autosomal chromosomal DNA. An unresolved question is how to discover candidate ICRs across the entire canine genome. Previously, bioinformatics analyses found a significant fraction of well-known ICRs in mouse, human, and *Bos taurus*. Analyses were based on finding the genomic positions of clusters of several CpG-rich motifs known as ZFBS-morph overlaps. These motifs are composite DNA elements. For this report, we performed similar studies to pinpoint candidate ICRs in the dog genome. A key feature of the bioinformatics approach is creating density plots to mark cluster positions as peaks. In genome-wide analyses, peaks in plots effectively discovered candidate ICRs along chromosomal DNA sequences of the *Canis familiaris* breed Boxer. With respect to Non-Dog RefSeq Genes, several candidate ICRs are in regions analogous to ICR positions in mouse DNA, in human DNA, or both. In the Boxer genome, examples include candidate ICRs for parent-of-origin-specific expression of the *MEST* isoform *PEG1*, *INPP5F_V2*, the *PLAGL1* isoform *ZAC1*, *IGF2R*, *PEG3*, and *GNAS* loci. In mouse, imprinted genes in these loci play important roles in developmental and physiological processes.

## Introduction 

In the genomic era, Man’s Best Friend offers the opportunity to identify genes that impact development, morphology, and behavior in *Canis familiaris* [1]. During normal embryonic development, genomic imprinting plays essential roles [2]. The process has evolved to regulate the expression of a subset of autosomal genes from either the maternal or the paternal allele [3]. Commonly, genomic imprinting affects viviparous and eutherians mammals [4]. Its impacts manifest broadly: at the level of specific cells, an organ, or a whole organism [5]. Genomic imprinting regulates gene dosage [4, 6]. Since numerous genes are normally expressed from both autosomal alleles, their dosage is two. For monoallelically expressed genes, the dosage is one. In genomic imprinting, a reduction in gene dosage results when specific DNA methyltransferases begin to modify the CpGs in ICRs in the maternally or paternally inherited allele [7, 8]. ZFP57 binds CpG modified hexameric sites (TGC ^me^CGC) to protect the DNA from demethylation [9–11]. Additional steps include recruitment of SETDB1 to trimethylate lysine 9 in histone H3, and HP1-mediated heterochromatin formation to completely silence imprinted genes [7].

The importance of genomic imprinting emerged from studies of mice, humans, and farm animals [12–14]. In mouse, ICRs correspond to a relatively small fraction of methylated DNA. They impact fetal and postnatal events [15]. In human, imprinting disorders manifest as developmental anomalies known as syndromes [16]. In *Bos taurus*, assisted reproductive technologies may lead to overgrowth syndromes: partly due to biallelic expression of several imprinted genes [17]. In mice, methylation of ICRs results in silencing a subset of genes [7]. In dogs, DNA methylation is linked to behavior, morphology and stranger-directed fear [18]. In the canines’ brain, allelic differences in DMRs affected domestication, breed formation, and behavior [19]. A noteworthy question is whether, in the course of domestication, new ICRs have emerged to repress gene expression in one of the two parental alleles. This scenario could lead to selection of many breeds or behavioral traits in dogs. Even though a review summarized likely impacts of genomic imprinting on dogs [20], progress in that area includes a relatively few publications [21, 22]. Thus, not much is known about imprinted genes in canines and the ICRs that regulate their allele-specific expression. For studies of parent-of-origin-specific expression, investigators begin with known imprinted genes or transcripts in mice; subsequently, they examine whether such transcripts are also monoallelically expressed in other species [21, 23–25]. After that step, they locate ICRs by devising various experimental techniques. However, one could imagine that finding an unknown imprinted gene or an ICR, one at a time, might be costly and unrealistic. Also, what phenotype to search for to identify an unknown imprinted gene? Could computational methods facilitate locating ICRs and imprinted genes across the entire genome for subsequent experimental validations? For mice, efforts in that direction include applying machine learning approaches to recognize candidate imprinted genes and to classify their parent of origin preference for expression [26]. Another approach combined computational and experimental methods to identify novel human imprinted genes [27]. What about dogs? Not much is known [20].

For studies of genomic imprinting, we developed a novel approach: first, locate ICRs genome-wide, then find the genes in their vicinity as potential imprinted genes. Our approach does not involve machine learning. It is simply based on the importance of the ZFP57 binding sequence to maintain the memory of allele-specific gene expression [10, 11, 28]. Notably for mice, our approach identified candidate ICRs for several potential imprinted genes with diverse functions including chromatin remodeling and regulation of gene expression [29]. For humans, it identified genes known for their association with developmental anomalies [30]. For *Bos taurus*, it identified potential imprinted genes known to impact growth, meat quality, milk production in cows, and spermatogenesis in bulls [31, 32]. Since not much is known about genomic imprinting in canines [20], in this report we explore whether our approach could also locate candidate ICRs in *Canis familiaris* genome-wide. To test this idea, we focus on candidate ICRs in the vicinity of genes known to be imprinted in mice, humane, or both.

## Results

###  A summary of our approach 

Since binding of ZFP57 to its methylated hexameric sites maintains allele-specific gene expression [9, 10], one may ask whether by selecting TGCCGC as a starting point, one could identify the well-known ICRs in mouse [33]. That question led to the discovery of a set of composite CpG-rich DNA elements [33, 34]. Since these elements consisted of TGGCGC overlapping a subset of MLL1 binding units––known as MLL1 morphemes [35]–– they were named ZFBS-morph overlaps [33]. For the sake of brevity, henceforth, we will refer to the composite DNA elements as ZMOs. To pinpoint cluster-positions, genome-wide, we created plots to display the density of ZMOs in a sliding window consisting of 850 bases. As other plots, we selected the midpoint of the boundaries (the window) on the X-axis —nucleotide positions. The Y-axis assesses the robustness of a peak from 0 to 5. Unpublished evaluations revealed that peaks that cover two ZMOs could be true or false positive.

### Datasets and their visualization

In publications, figures present results of investigations. Although informative, figures do not effectively demonstrate the power of our approach. In fact, visualization is essential for data-driven research discoveries [36]. To do so, we formatted our datasets for dynamic visualization on the UCSC genome browser. Based on this approach, on the browser, we created two custom tracks to display the positions of TGCCGC hexamer and ZMOs. Another custom track marks nucleotide positions of peaks in density plots. On the genome browser, one can assess the scale, specificity, or robustness of the approach. In density plots, the X-axis gives nucleotide positions. The Y-axis assesses the robustness of a peak. It gives a likelihood estimation from 0 to 5. Robustness varies according to the numbers of ZMOs under a peak: 5 is more robust than 4, 4 is more robust than 3, 3 is more robust than 2. Furthermore, on the genome browser [37], researchers can dynamically view our datasets with respect to genomic landmarks including RefSeq Genes, CpG islands, and Non-Dog RefSeq Genes [38].

### A candidate ICR in deduced *MEST* locus in Boxer

During mouse development, *Mest* is expressed biallelically in mesodermal derivatives and the hypothalamus [39, 40]. In mouse, the *Mest* locus encompasses an intragenic promoter for regulating parent-of-origin-specific expression of Peg*1*, the *Mest* imprinted isoform, and a promoter for driving transcription of biallelically-expressed gene [40]. Human DNA, in addition to the isoform-specific *MEST* transcript, encompasses another imprinted gene (*MESTIT1*) that specifies a noncoding antisense RNA [41, 42].

**Fig. 1:**
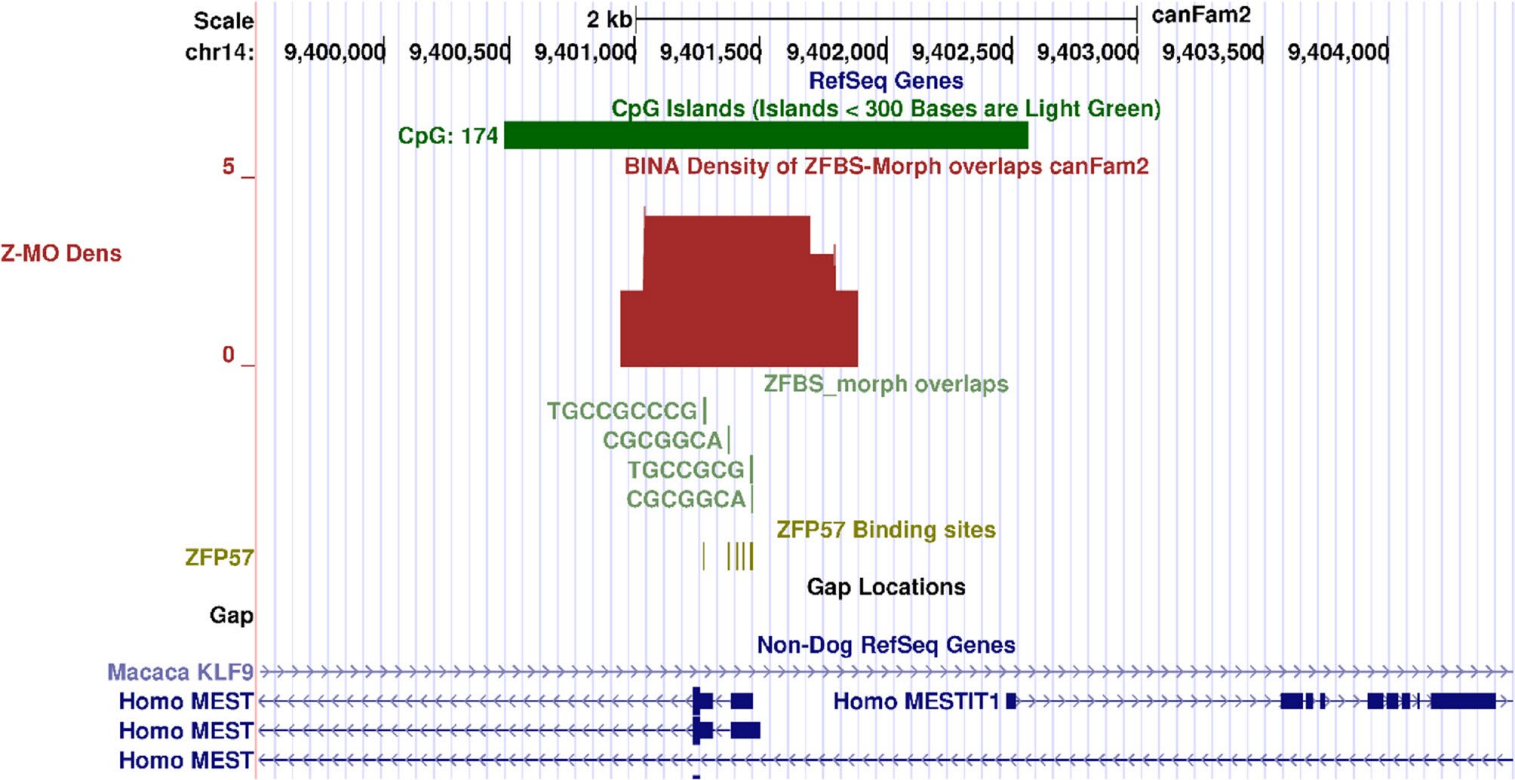
Candidate ICR for allele-specific expression of *MEST* isoform (*PEG1*) in the Boxer genome. On the genome browser, genomic landmarks are displayed on horizonal lines known as tracks. A track, colored green, gives the positions of CpG islands in the Boxer genome. A custom track, maroon color, displays peak position in density plots. In hunter green are tracks for viewing the positions of ZFBS-morph overlaps (ZMOs), in pack format, and ZFP57 hexamers in dense format. Tracks in dark blue displays Non-Dog RefSeq Genes. In density-plots, a peak located a candidate ICR in a CpG island (CpG174)

RefSeq database maintains and analyzes the sequences of annotated genes, transcripts, and proteins [43]. These records are for annotating gene-positions across genomic DNA. Since the Boxer genome is sparsely annotated, the UCSC browser displays gene-position with respect to Non-Dog RefSeq (Fig. 1). For example, with respect to human RefSeq genes, the Boxer genome includes transcripts labelled Homo MEST and Homo MESTIT1 (Fig. 1). In closeup views, two transcripts are intronic. Keeping in mind reported studies [41, 42, 44], for Boxer, one could infer the following scenarios. The longest annotated *MEST* transcript is biallelically expressed, and the shorter ones are imprinted transcripts (Fig. 1). Our approach predicts a candidate ICR that agrees with both scenarios. Specifically, in Boxer DNA, the density plots include a peak (a candidate ICR) in the intron of the annotated long *MEST* transcript. This intronic peak is in a CpG island (CpG174). It encompasses the transcription start sites (TSSs) of the shorter *MEST* transcripts (Fig. 1). Near the peak is the TSS of MESTIT*1*. Therefore, our approach located a candidate ICR for parent-of-origin-specific expression of both *PEG1* and MESTIT*1* in canine DNA (Fig. 1).

In asexual reproduction (parthenogenesis), if the maternal gene is imprinted, the absence of a paternally expressed gene often causes embryonic lethality [39]. However, genomic targeting experiments could introduce a mutation in *Peg1* to create viable and fertile female mice [45]. Due to loss of *Peg1*, these females displayed unusual behavior [45]. Their response could be relevant to studies of canines. Initially, the mice appeared normal: they delivered at term with a normal pregnancy rate [45]. They also showed an initial normal investigative behavior towards their pups. However, they did not demonstrate the expected maternal response to feeding and nurturing [45]. Furthermore, viable females displayed impaired placentophagia: a partly nutritional behavior observed in many mammals [45].

### A candidate ICR in deduced PLAGL1 locus in Boxer

**Fig. 2:**
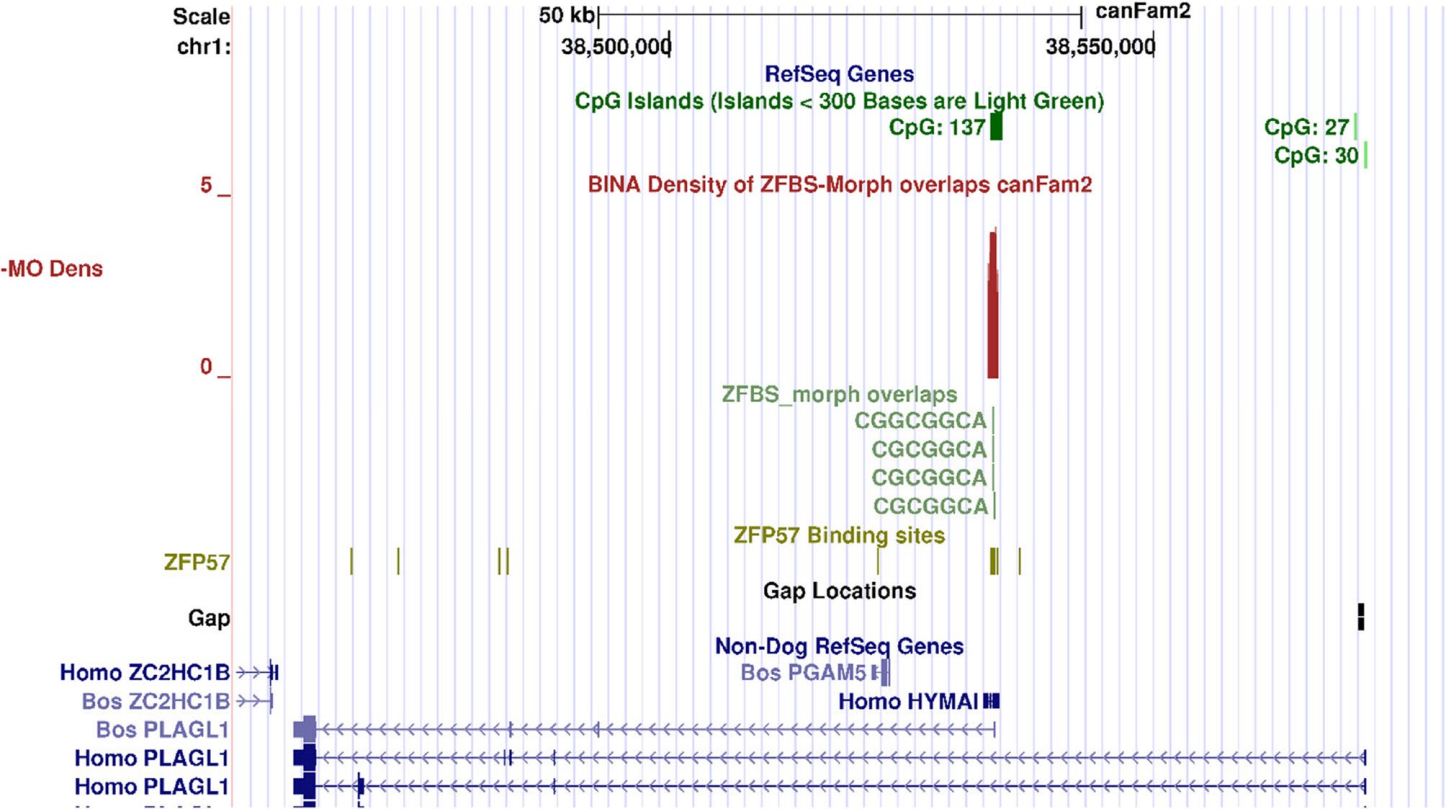
Candidate ICR for allele-specific expression of *PLAGL1* isoform (*ZAC1*) in the Boxer genome. From top to bottom: the positions of CpG islands, in pack format; density-plot, in full format; ZFBS-morph overlap (ZMOs), in pack format; ZFP57 hexameric sites, in dense format; and Non-Dog RefSeq Genes, in pack format. The candidate ICR is marked by a robust peak in CpG137

According to nucleotide databases, PLAGL1 related proteins are translated from several related mRNA isoforms. Members of the PLAGL1 family bind DNA to regulate gene expression [46]. Human *PLAGL1* locus encompasses an intronic CpG island that includes the initiation site for a maternally expressed transcript known as *LOT1* or *ZAC1* [47]. Occasionally, *ZAC1* is referred to as *PLAGL1* [47, 48]. In addition to *ZAC1*, human DNA includes another imprinted intronic gene (*HYMAI*) transcribed to produce a noncoding RNA [49]. With respect to Non-Dog RefSeq genes, Boxer DNA includes several *PLAGL1* transcripts (Fig. 2). As reported for human and mouse [47, 48], the locus in Boxer also contains an intronic CpG island (CpG137). This island includes the TSS of both *HYMAI* and *ZAC1*, referred to as PLAGL*1* (Fig. 2). Density plots include a robust peak in CpG137. Therefore, our approach correctly identified a candidate ICR for imprinted expression of both *ZAC1* and *HYMAI* in Boxer DNA (Fig. 2).

### A candidate ICR in deduced *INPP5F* locus in Boxer

Also known as SAC2, INPP5F is a member of the inositol polyphosphate-5-phosphatase family of enzymes [50]. In both mice and humans, nearly all INPP5F transcripts are produced biallelically from a relatively long gene consisting of many exons and introns [51]. In combination with chromosome anomalies in mouse, tissue-specific microarray screening identified an imprinted transcript (*Inpp5f_v2*) expressed from the paternal allele in the brain [51]. This transcript has a unique alternative first exon [51]. Its TSS is within an intronic CpG island that includes the ICR for repressing *Inpp5f_v2* expression from the maternal allele [51].

**Fig. 3:**
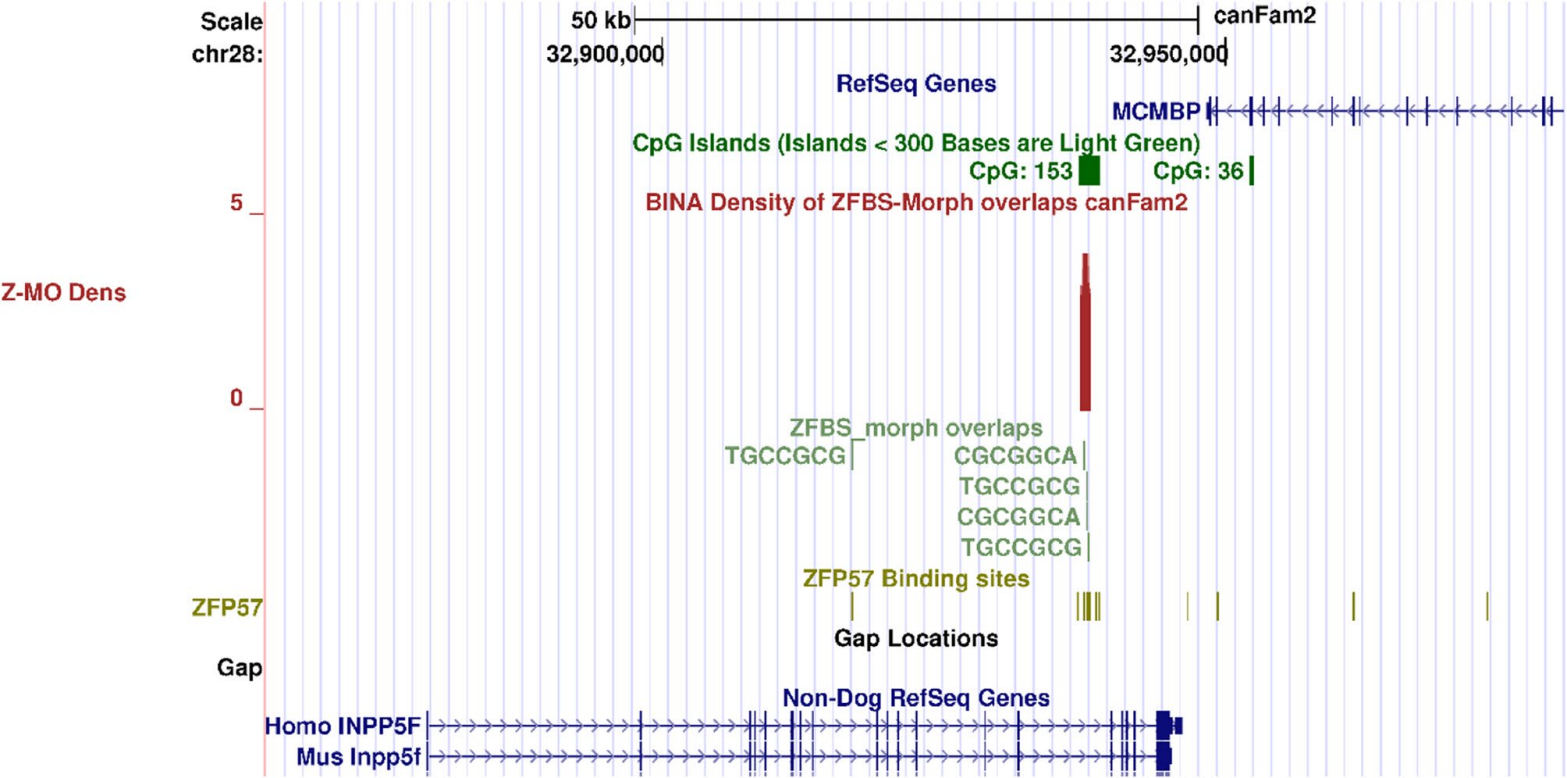
Candidate ICR for allele-specific expression of *INPP5F_V2* in the Boxer genome. From top to bottom: the positions of CpG islands; density plot; ZFBS-morph overlaps (ZMOs); ZFP57 hexameric sites; and Non-Dog RefSeq Genes. The candidate ICR is marked by a robust peak in CpG153. In the context of studies of mice and humans, the ICR is intronic and at a correct position

Similarly, with respect to Non-Dog RefSeq Genes, the Boxer genome includes an intronic CpG island (CpG153). In density plots, a peak is evident within that island. In the context of *Inpp5f_v2* TSS in mouse [51], the peak in CpG153 correctly marks the position of a candidate ICR for imprinted *INPP5F_V2* expression in Boxer DNA (Fig. 3).

### A candidate ICR in deduced *ZIM2/PEG3* locus in Boxer

In mouse, *Peg3* regulates fetal growth and maternal behavior towards her pups [52]. The *Peg3* imprinted domain encompasses several genes expressed from the paternal allele; a few genes are expressed from the maternal allele [53]. A single CpG-rich imprinted gDMR regulates parent-of-origin-specific expression of the entire *Peg3* imprinted domain [53]. In mouse, the TSSs of *Zim*2 and *Peg3* are at differing positions; in human, transcription start sites of *ZIM*2 and *PEG3* coincide [53]. As in mouse, human *PEG3* is imprinted on the maternal allele [53].

**Fig. 4:**
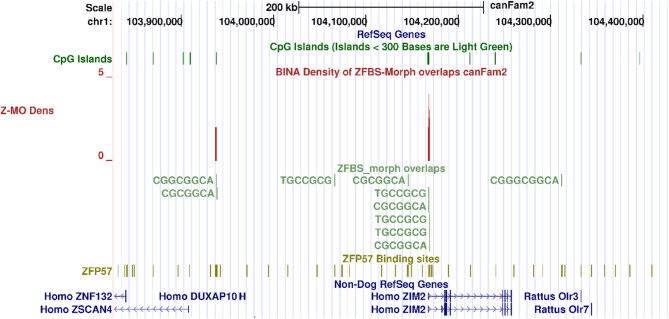
Candidate ICR for allele specific expression of *PEG3*/*ZIM2* in the Boxer genome. From top to bottom: the positions of CpG islands; density plot; ZFBS-morph overlaps (ZMOs); ZFP57 hexameric sites; and Non-Dog RefSeq Genes. The robust peak marks the candidate ICR location. In the context of studies of humans, candidate ICR position is correct

As observed in mouse [53], in the Boxer genome, a relatively long DNA segment encompasses several CpG islands. With respect to human RefSeq Genes, Boxer includes a gene annotated as Homo ZIM2 (Fig. 4). In Boxer, our approach identified a candidate ICR for allele-specific expression of *PEG3/ZIM*2. In density plots, the candidate ICR is defined by a peak in the CpG island that encompasses the first exon of the transcript annotated as ZIM2 (Fig. 4). Therefore, in Boxer DNA, this peak correctly located a candidate ICR for imprinted expression of *ZIM2*/*PEG3*.

### A candidate ICR in deduced complex *GNAS* locus in Boxer

The *GNAS* locus consists of several related transcripts expressed from different promoters [54]. Its complexity stems from differing promoters and pre-mRNA splicing events producing related transcripts [54]. Among *GNAS* transcripts, *Gαs* is biallelically produced; *XLαs* is expressed from the paternal allele [54]. Both *Gαs* and *XLas* specify related G-proteins with shared and distinguishable properties [55]. G-protein-coupled receptors (GPCRs) respond to extracellular inputs to relay information across outer cellular membrane to evoke proper physiological outcomes [56]. Downstream effects of a GPCR depends on which G protein type(s) it is coupled with [57].

**Fig. 5:**
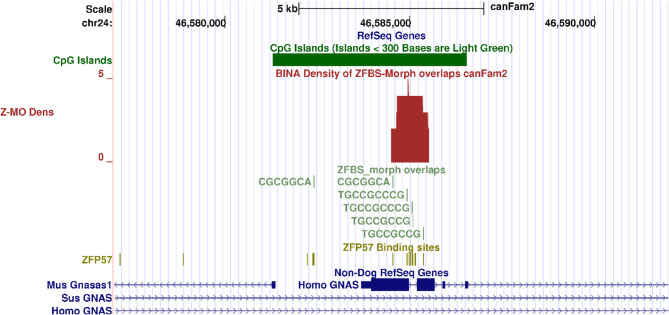
Candidate ICR in the *GNAS* complex locus in the Boxer genome. From top to bottom: the positions of CpG islands; density plot; ZFBS-morph overlaps; ZFP57 binding sites (ZMOs); and Non-Dog RefSeq Genes. The robust peak marks the likely position of a candidate for principal ICR in *GNAS* complex locus in Boxer

In mice, a principal ICR regulates allele-specific expression of several *GNAS* transcripts [58]. Since *XLas* encodes a G-protein, it is essential to identifying the ICR that regulates its parent-of-origin-specific expression. In Boxer DNA, a search located the complex *GNAS* locus with respect to human RefSeq Genes (Fig. 5). Examination of density plots revealed a peak in a CpG island. Since this peak encompasses *XLas* TSS, it offers a candidate ICR for the complex *GNAS* locus in Boxer DNA (Fig. 5).

### A candidate ICR in *IGF2R* locus in Boxer

**Fig. 6:**
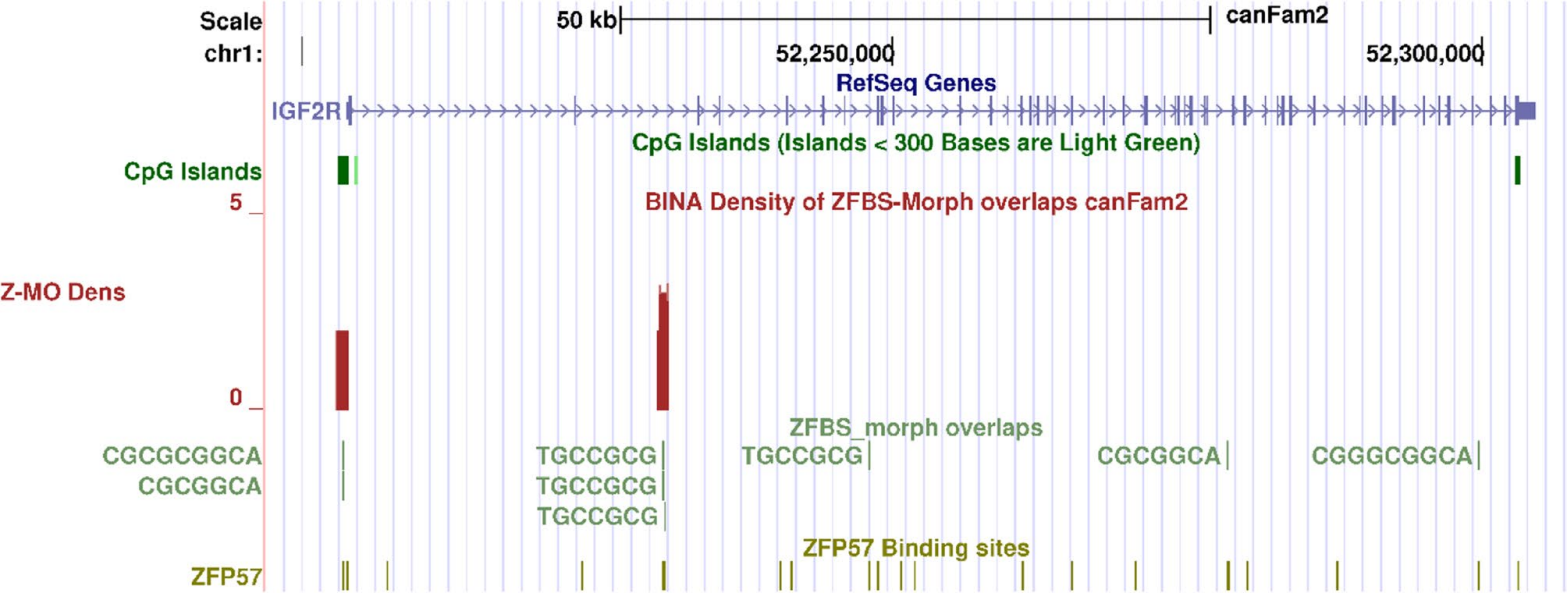
Candidate ICR for allele specific expression of *IGF2R* in the Boxer genome. From top to bottom: the positions of CpG islands; density plot; ZFBS-morph overlaps (ZMOs); ZFP57 hexameric sites; and Non-Dog RefSeq Genes. The candidate ICR is intronic and marked by a robust peak

In mouse, the *Igf2r* imprinted domain encompasses at least two DMRs [59]. Even though the DMR at the *Igf2r* promoter is methylated on the repressed paternal allele, it does not exhibit expected characteristics of an ICR [59]. The other DMR is a domain-wide ICR [59]. In mouse, the ICR is in the second *Igf2r* intron and is encompassed by a CpG island [60]. The intronic DNA also includes the TSS of an imprinted noncoding RNA gene known as *Airn* [59]. With respect to *Igf2r*, *Airn* is in an antisense orientation [59]. Evidence suggests a role for *Airn* in regulating imprinted *Igf2r* expression [59]. However, in dogs, *IGF2R* was expressed monoallelically even in the absence of *AIRN* [21]. Our approach predicted a candidate ICR in the second intron of *IGF2R* in Boxer DNA (Fig. 4). Its position agrees with the intronic ICR position in mouse [60]. It also agrees with the ICR position in *Canis familiaris* [21]. In dog, the ICR is in a CpG island [21]. However, on the browser, there is no CpG island in the second *IGF2R* intron (Figs. 4 and 6).

### Genomic imprinting of *H19 *– *IGF2* domain in *Canis familiaris*

**Fig. 7:**
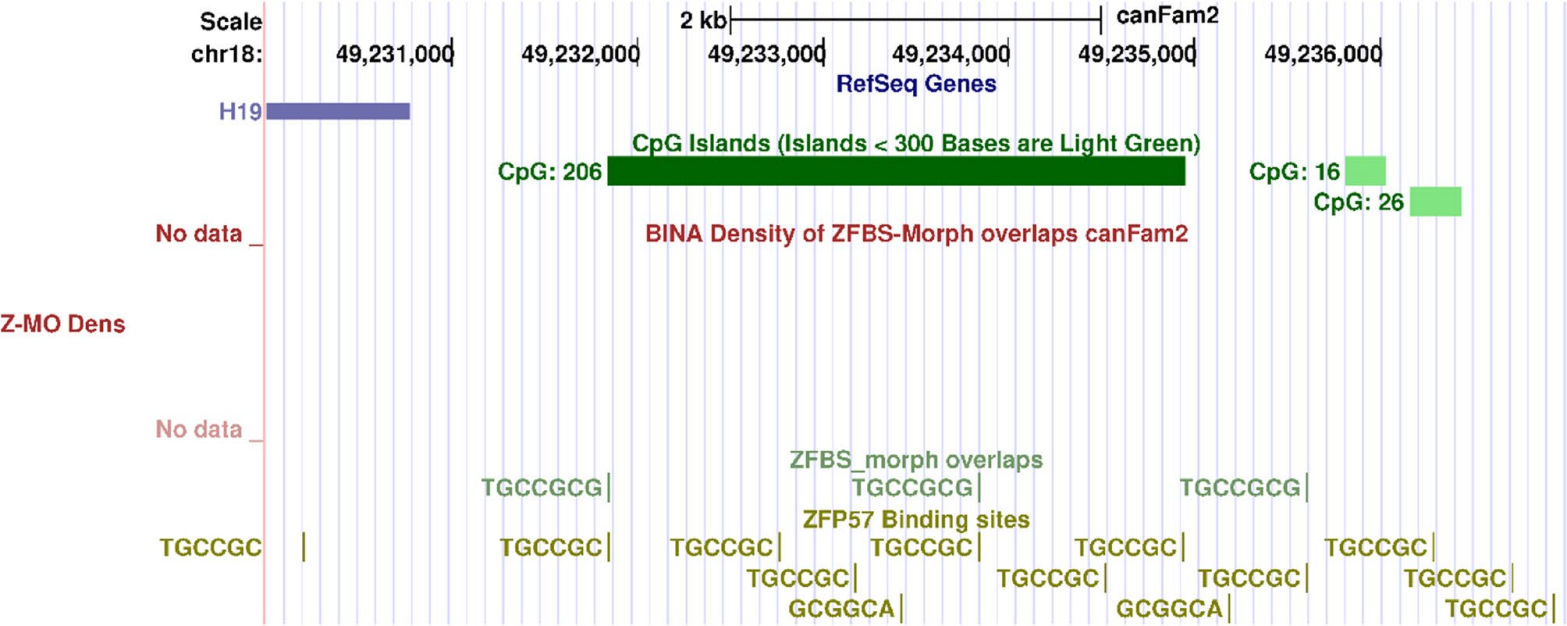
*H19 *locus in Boxer genome. From top to bottom: the positions of H19, CpG islands, ZFBS-morph overlaps (ZMOs), and hexameric sequence. Upstream H19, the approach did not find a candidate ICR for regulating allele-specific expression in Boxer DNA. the region includes 3 ZFBS-morph overlaps (ZMOs), and several hexameric site

In canine, sequences upstream of *H19* encompass reiterated DNA [22]. They include two direct repeats with partial internal repeats. The domain encompasses a putative differentially methylated ICR with likely paternal transmission [22]. Previously, our approach correctly identified the ICR of the *IGF2*–*H19* imprinted domain in mouse [29], in human [30], and in *Bos taurus* [31]. However, since the approach relies on detecting clusters of ZMOs, it did not predict a candidate ICR in Boxer DNA (Fig. 7). We find that upstream of *H19*, the Boxer genome encompasses three ZMOs, and several hexameric sequences that after methylation, bind ZFP57 (Fig. 7). In mouse ESCs, ZFP57 binding to methylated hexameric sites in ICRs was required to maintain allele-specific expression [9]. Therefore, an intriguing question is whether ZFP57 also contributes to the regulation of the *IGF2*–*H19* domain in canine DNA.

## Discussion

In eutherian mammals, genomic imprinting regulates parent-of-origin-specific expression [61]. Therefore, as in other mammals, imprinted genes could also influence embryogenesis and fetal development in *Canis familiaris* [20]. Since only a few studies have addressed genomic imprinting in dogs [20, 22, 62], locating candidate ICRs in Boxer genome might help with identifying potential allele-specific expressed genes with possible impact on canine embryonic development, and postnatal behavior and physiology.

Since the Boxer genome includes chromosomal anomalies [63], we were unsure whether our approach would work for studies of genomic imprinting in *Canis familiaris*. Along Boxer genome, the approach succeeded with identifying candidate ICRs for allele-specific expression of several well-known imprinted genes in mice, humans or both, including: *PEG1*/*MEST* isoform, *PLAGL1*/*ZAC1* isoform, *INPP5F_V2* isoform, *ZIM2*/*PEG3*, *IGF2R*, and *GNAS XLαs* (Figs. 1, 2, 3, 4, 5 and 6). In mouse, the *Mest* isoform *Peg1* impacts maternal response to her newly born pups [45]. Dog pups also need maternal interactions for survival, nourishment, and protection. However, not much is known about genes that regulate maternal behavior in canines [64].

During embryogenesis in mice, a well-known imprinted transcript (*Zac1*) is expressed in the progenitor/stem cells of several tissues including neuronal and skeletal muscle [65]. ZAC1 functions include regulation of cell-specific expression of a G protein-coupled receptor, PAC1R, with important roles in signaling pathways [66]. In response to pituitary adenylate cyclase activating polypeptide (PACAP), PAC1R transmits signals to the neuroendocrine, endocrine, and nervous systems [66, 67]. In dog ileal circular muscle, PACAP operated as a neurotransmitter [68]. In mouse, *Inpp5f_v2* is expressed in the brain [51]. Mutations in another allele-specific gene (*Peg3*) produced maternal behavioral defects [52]. Their abnormal behavior could be due to anomalies in oxytocin signally pathway [52]. Known as “the love hormone” [69], oxytocin mediates dogs’ human-directed social behavior [70]. In the *Gnas* complex locus, one of the imprinted transcripts (*XLαs*) encodes a G-protein that functions in signaling pathways by several GPCRs [71]. *XLαs* expression was detected in distinct regions of the midbrain, the hindbrain, the noradrenergic system of the brain, and in neuroendocrine tissues [54, 72]. Furthermore, XL*αs* is crucial for early postnatal adaptation to feeding and survival [54]. Lastly, IGF2R (insulin-like growth factor 2 receptor) facilitates IGF2 endocytosis [20].

### What about species differences among candidate ICRs?

To explore the possibility of species differences among candidate ICRs, we randomly selected a few candidate ICRs to assess the function of genes in their vicinity. In *Bos taurus*, a robust peak was found in the vicinity of *HMGA2* [31]. Polymorphisms in *HMGA2* impacted height in human and body stature in cattle. This gene also influenced body size in mice. Our approach identified *HMGA2* as a potential imprinted gene in *Bos taurus*, humans, and mice. Our approach predicts that *HMGA2* is not a potential imprinted gene in Boxer. A notable possibility is the emergence of new ICRs during animal domestication. Examples include *HELB*, a gene that encodes a DNA helicase with important functions in DNA replication and stress responses to DNA damage [73]. In *Canis familiaris*, a very robust candidate ICR is upstream of *HELB* TSS. Since in Bos *taurus*, a robust peak is at a similar position, *HELB* is also a potential imprinted gene in cattle. Our approach predicts that *HELB* is not a potential imprinted gene in humans nor in mice. Concerning dog breeds: in studies of signatures of selection, *HELB* was among the genes associated with dog size in Italian livestock guardian and herding shepherd dogs [74]. Furthermore, in the Boxer genome, exploratory analyses identified a candidate ICR for allele-specific expression of one of the *RBPJ* transcriptional isoforms. This gene encodes a transcription factor that mediates signal transmission by Notch receptors [75]. These preceptors are extracellular proteins involved in controlling cell fate decisions and patterning, and in the maintenance of muscle progenitor cells during embryogenesis [75].

## Conclusion

Our study reveals that as in other mammals, the genome of *Canis familiaris* is likely to encompasses ICRs for parent-of-origin-specific expression. With dynamic visualization on the UCSC genome browser, researchers can discover candidate ICRs for potential novel imprinted genes across the entire Boxer genome.

## Methods

Underlying hypothesis: If genomic DNA is ‘the book of life’, then it should contain words for conveying information. Codons are well-known and authenticated examples. Since the binding of ZFP57 to methylated TGCCGC maintains allele-specific expression [9–11], TGCCGC is a word in mammalian DNA. Perl programming was developed to handle documents consisting of text. Since text in DNA is written with 4 chemical alphabets (A G C T), Perl scripts are suitable for locating sequence motifs (words) in DNA.

### Marking the genomic positions of ZFP57 hexameric site and ZMOs

To locate the hexameric sequence (TGCCGC), genome-wide, we wrote a Perl script. This program scanned a specified Boxer chromosomal DNA. Its output consisted of nucleotide positions of TGCCGC along the DNA. The English language includes compound words. Genomic DNA includes composite words, for example: the composite DNA elements known as ZFBS-morph overlaps or ZMOs [33]. Based on this idea, a Perl script located the positions of ZMOs along chromosomal DNA. Subsequently, we wrote UNIX subroutines, to combine both outputs to create a dataset suitable for upload on the UCSC genome browser.

### Design of density plots for candidate ICRs localization

In genomic DNA, clustering of words reflects the presence of regulatory regions [76].

In books, words in sentences are context-dependent and appear in various orders and combinations. Therefore, it is important to capture the context of regulatory motifs in regulatory regions of genes [33, 35, 76, 77]. In ICRs, ZMOs appear various orders and combinations [29–33]. In enlarged views, one can easily see the genomic positions of ZMO clusters (Figs. 1, 2, 3, 4, 5 and 6). However, visual inspections cannot find cluster positions in long DNA segments or across an entire length of chromosome DNA. As a solution to this problem, we created density plots. We wrote another Perl script to scan the files containing genomic positions of ZMOs. This script opens the file for a specified chromosome where ZMOs positions were determined. Subsequently, the Script starts at about nucleotide 6. Afterwards, the script begins reading the chromosomal DNA sequence while moving along the DNA using a predefined window. By trial and error, we chose a window consisting of 850 nucleotides, as detailed in [29]. To save computation time, the window moved along the DNA six nucleotides at a time. In each window, the script initially checked whether it contained any ZMOs. If the window encompassed 2 or more ZMOs, then, in an output file, the program recorded the nucleotide position at the midpoint of each window, on the X-axis, the number of ZMOs on the Y-axis. In computed plots, various methods usually select a midpoint position. The sliding window stops when it reaches a few bases before the end of chromosomal DNA. The last step consisted of writing UNIX subroutines to combine and to tailor the outputs for display as a custom track on the UCSC genome browser.

## Data Availability

Datasets can be accessed for download from Purdue University Research Repository (PURR). - The positions of ZFBS hexamers and ZFBS-morph overlaps in the build canFam2 of the dog genome https://purr.purdue.edu/publications/3519/1 - Density of ZFBS-morph overlaps in the build canFam2 of the dog genome https://purr.purdue.edu/publications/3520/1 With practice, one can easily learn how to navigate and use track controls on the genome browser. To navigate, it is important to use a computer attached or in syn with a mouse. Key features of the browser include ‘a box’ for typing a query to find a gene (for example, MEST). If MESTis an annotated RefSeq gene, the browser will display its genomic position in Boxer DNA. If not, to locate MEST, one could select the track that displays Non-dog RefSeq genes. The browser includes pulldown menus for viewing a track in dense, pack, or dense format. For long chromosomal DNA segments, select dense. For closeup views, select pack. The density plots should always be displayed in full format. The browser also provides arrows for zooming in or zooming out. For long DNA segments, the easiest way to zoom in is to use your computer mouse to frame the region of interest. While producing the datasets, CanFam2 was the latest available build of the Boxer genome.

## Abbreviations

gDMR: gametic or germline differentially methylated region
ICR: Imprinting control region
TSS: Transcription start site
ZMOs: ZFBS-morph overlaps are composite DNA elements
